# Cigarette pack size and consumption: a randomized cross‐over trial

**DOI:** 10.1111/add.16062

**Published:** 2022-11-03

**Authors:** Ilse Lee, Anna K. M. Blackwell, Alice Hobson, Danielle Wiggers, David Hammond, Katie De‐loyde, Mark A. Pilling, Gareth J. Hollands, Marcus R. Munafò, Theresa M. Marteau

**Affiliations:** ^1^ Behaviour and Health Research Unit University of Cambridge Cambridge UK; ^2^ School of Psychological Science University of Bristol, 12a Priory Road Bristol UK; ^3^ Nuffield Department of Primary Care Health Sciences University of Oxford Oxford UK; ^4^ School of Public Health Sciences, Faculty of Health University of Waterloo Waterloo Ontario Canada; ^5^ EPPI Centre, UCL Social Research Institute University College London London UK

**Keywords:** cigarettes, Cigarette pack size, randomised cross‐over trial, smoking, tobacco control, tobacco control policy

## Abstract

**Background and Aims:**

Smoking fewer cigarettes per day may increase the chances of stopping smoking. Capping the number of cigarettes per pack is a promising policy option, but the causal impact of such a change is unknown. This study aimed to test the hypothesis that lowering cigarette pack sizes from 25 to 20 reduces the number of cigarettes smoked.

**Design:**

This randomized controlled cross‐over trial had two 14‐day intervention periods with an intervening 7‐day period of usual behaviour. Participants purchased their own cigarettes. They were instructed to smoke their usual brand from either one of two sizes of pack in each of two 14‐day intervention periods: (a) 25 cigarettes and (b) 20 cigarettes. Participants were randomized to the order in which they smoked from the two pack sizes (a–b; b–a).

**Setting:**

Canada.

**Participants:**

Participants were adult smokers who smoked from pack sizes of 25, recruited between July 2020 and June 2021. Of 252 randomized, 240 (95%) completed the study and 236 (94%) provided sufficient data for the primary analysis.

**Measurements:**

Cigarettes smoked per participant per day.

**Findings:**

Participants smoked fewer cigarettes per day from packs of 20 cigarettes [*n* = 234, mean = 15.7 standard deviation (SD) = 7.1] than from packs of 25 (*n* = 235, mean = 16.9, SD = 7.1). After adjusting for pre‐specified covariates (baseline consumption and heaviness of smoking), modelling estimated that participants smoked 1.3 fewer cigarettes per day [95% confidence interval (CI) = −1.7 to −0.9], equivalent to 7.6% fewer (95% CI = −10.1 to −5.2%) from packs of 20 cigarettes.

**Conclusions:**

Smoking from packs of 20 compared with 25 cigarettes reduced the number of cigarettes smoked per day.

## INTRODUCTION

Despite the global fall in prevalence of tobacco smoking in the last three decades, the growth in population has increased the number of smokers from 0.99 billion in 1990 to an all‐time high of 1.14 billion in 2019 [1]. It remains one of the largest risk factors for disease globally, rated in 2019 as the second most important risk factor for all ages [2] and a major cause of the gap in life expectancy and years in good health between the richest and poorest groups [2].

The size of cigarette packs—the number of cigarettes in a single pack—is a potentially important but neglected tobacco control target [3, 4]. However, the impact of cigarette pack size on smoking is uncertain. There are two key uncertainties. First, does smoking from a smaller pack size reduce the number of cigarettes smoked? Secondly, does smoking fewer cigarettes per day increase the chances of subsequently stopping smoking? The aim of the current study is to address the first uncertainty.

An increasing number of jurisdictions have set a minimum size of 20 cigarettes per pack to make cigarettes less affordable to young people, in accordance with the international recommendations under the WHO Framework Convention on Tobacco Control [3, 4, 5, 6, 7, 8]. Packs of 20 cigarettes are standard in most countries, although larger pack sizes are common in some countries such as Canada and Australia [3]. In an earlier parallel group study using a two‐stage adaptive design we randomized smokers in Australia, who usually purchased cigarettes in packs of at least 25, to smoke for 4 weeks either from their usual pack size or from packs of 20 cigarettes. At the interim assessment stage (when 124 participants had been randomized) this adaptive trial was halted as the estimated required total sample of more than 1000 exceeded pre‐specified criteria for feasible recruitment [9]. A cross‐over design, making within‐group comparisons, was deemed more efficient, as these typically require smaller numbers of participants.

Based on robust evidence from studies of food consumption [10], it is plausible that smaller pack sizes reduce cigarette consumption. Limited, non‐experimental evidence suggests an association between cigarette pack size and consumption. For example, those smoking more heavily tend to purchase cigarettes in larger packs [11]. In a more recent study based on a hypothetical purchase task, smokers wanting to self‐regulate their consumption reported a preference for smaller packs [12].

The causal nature of the association between pack size and cigarettes smoked per day remains uncertain. The aim of the current randomized controlled cross‐over trial is to assess the impact on cigarette consumption of using packs of 20 cigarettes compared to packs of 25 cigarettes.

## METHODS

The study was approved by the University of Cambridge Psychology Research Ethics Committee (PRE.2019.068) and the University of Waterloo Research Ethics Board (#41353). Participants provided written informed consent to participate.

The study protocol was prospectively registered with ISRCTN on 6 March 2020 (ISRCTN16013277) and the Open Science Framework (OSF) on 21 November 2019, updated on 9 July 2020 (https://osf.io/zby94). Recruitment was planned to start in March 2020, but was delayed until July 2020 due to the COVID‐19 pandemic. The statistical analysis plan was uploaded to both OSF and ISRCTN on 20 July 2021 prior to data analysis.

### Study design

The study was a randomized controlled cross‐over trial with two conditions—pack size of 20 cigarettes versus pack size of 25 cigarettes—separated by a period of ‘usual behaviour’.

### Participants

A total of 252 smokers in Canada participated in the study from July 2020 until June 2021, recruited by a research agency (https://leger360.com/). In Canada cigarettes are sold in two pack sizes, 20 and 25. Most sales (71%) are for the larger pack sizes [13]. Eligible participants were those with the following characteristics:

- aged 19 years and over
- smoked factory‐made cigarettes
- had smoked at least 100 cigarettes in their life‐time
- currently smoked 10 or more cigarettes a day on every day of the week
- normally purchased cigarettes in packs of 25
- used a brand or brand variant in which cigarettes were available in pack sizes of 20 as well as 25 in a shop convenient to them
- lived anywhere in Canada outside British Columbia, Northwest Territories, Nunavut and Yukoni 1
- able to read and write sufficient English to complete all study procedures
- willing to record on each cigarette pack dates when the pack was opened and when finished
- willing to send photographs for 4 weeks of their completed cigarette packs
- willing to purchase and smoke their usual brand variant in packs of 20 for 2 weeks
- were not pregnant or trying to become pregnant
- were not intending to quit smoking in the next 3 months
- had not used e‐cigarettes at least weekly over the past month and intended to continue not doing so
- had not smoked one a week or more roll‐your‐own cigarettes over the past month and intended to continue not doing so
- did not normally transfer cigarettes into a case
- usually buy their own cigarettes
- did not live in the same household as someone who has enrolled in the study.

### Intervention

Participants were instructed to smoke their usual brand variant and length (king size or regular) of cigarettes from a single size of cigarette pack in each of the two intervention periods: (a) 20 cigarettes and (b) 25 cigarettes. Each intervention period lasted 14 days. Participants could smoke cigarettes of any brand variant and from either pack size in the ‘usual behaviour’ period, which lasted at least 7 days between the intervention periods. 2 This is classified as a size × product intervention within the typology of interventions in proximal physical micro‐environments (TIPPME) [14].

### Procedure

Participants were recruited and screened for eligibility by a research agency (https://leger360.com/), which sent a screening questionnaire based on the inclusion and exclusion criteria to 84 873 members of their research panel known to be smokers or on whom there was no information regarding their smoking status. Those who passed the screen were sent the study information sheet and invited to participate. The research agency referred 538 potential participants to the research team in total.

The study was presented as investigating how cigarette pack size affects the effectiveness of health warnings. This was to reduce the chance of participants focusing on their cigarette consumption in relation to pack size. Potential participants provided information on their demographic characteristics and smoking behaviour. Potential participants were asked to purchase one pack of 20 and one pack of 25 cigarettes in their usual brand variant. They sent photographs of these packs and their receipts to the research team. This was to check the eligibility criterion that they used a brand or brand variant available to buy in both pack sizes from a shop convenient to them.

#### Randomization

Allocation of participants to the order in which they completed the conditions was determined using a computer‐generated random number sequence prepared by one of the project statisticians (R.M.), using Stata version 15 (StataCorp LLC, TX, USA). Block randomization was used to generate an equal number of participants allocated to each treatment order and to reduce the potential of selection bias compared to simple randomization. The blocks were in sizes of 2, 4 and 6. The random number sequence, with IDs for the sequence of potential participants, was concealed from the research team, research agency and participants until the participant had consented to take part in the study and shown that they were able to purchase their usual brand variant of cigarettes in packs of 20 and packs of 25. When a participant was deemed eligible for randomization the research team accessed the next random allocation in the sequence, and this participant was assigned the corresponding ID. Instructions (see Supporting information, Material 1) were e‐mailed and mailed to participants, together with a set of stickers to attach to all the cigarette packs from which they would smoke during the intervention periods.

The stickers had space to record the following information: (i) participant ID; (ii) pack number; (iii) date the pack was finished; (iv) a rating from 1 to 7 for the effectiveness of the warning label (to bolster the credibility of the cover story); (v) number of cigarettes smoked themselves from the pack; (vi) number of cigarettes smoked from other non‐study packs while this study pack was open (e.g. given to them by a friend); and (vii) number of cigarettes remaining (for partially empty packs at the end of a study period).

On day 1 of each intervention period, participants were e‐mailed a reminder to only smoke their usual cigarette brand variant of cigarettes from packs of the appropriate size, as randomized.

On day 7 of each intervention period, participants sent photographs of all the cigarette packs they had finished since day 1. After smoking the last cigarette on day 14, participants sent photographs of all the cigarette packs they had smoked from since day 7, including any partially empty packs.

At the end of each intervention period, participants completed questionnaires about their smoking and cigarette purchasing during the study. Participants received Can$400 for completing the study procedures, and purchased their own cigarettes during the study.

### Measures

#### Primary outcome

The primary outcome was the mean number of cigarettes smoked per participant per day during each intervention period, calculated by dividing the total number of cigarettes smoked during each intervention period by 14. The number of cigarettes smoked during each intervention period was calculated from photographs taken by participants of their cigarette packs labelled with study stickers.

#### Secondary outcome

Motivation to stop smoking was measured using the single‐item Motivation to Stop Scale [15] with the question: ‘Which of the following describes you?'. Responses range from (1) ‘I don't want to stop smoking’ to (7) ‘I REALLY want to stop smoking and intend to in the next month'. Participants answered this question at the end of each intervention period.

#### Baseline smoking characteristics

Three measures were assessed at enrolment: heaviness of smoking (using the Heaviness of Smoking Index, HSI [16]), motivation to stop smoking (using the MTSS [15]) and self‐reported number of cigarettes smoked per day. The quantity of cigarettes participants tended to purchase at any one time was also recorded.

Demographic characteristics recorded at baseline included gender, age, household income, level of formal education and ethnicity.

#### Other measures

Participants answered questions in an on‐line questionnaire at the end of each intervention period to report any mitigating factors that they felt had affected their smoking in the preceding 2 weeks (see Supporting information, Material 2 for the list of questions).

Participants answered questions at the end of the study in an on‐line questionnaire covering their purchasing of cigarettes during each intervention period; that is, whether they bought cigarettes in single packs, multiple packs or cartons), reasons for pack size preferences and what they thought the study was about (see Supporting information, Material 2 for the list of questions).

### Sample size estimation

The within‐person standard deviation (SD) of cigarettes smoked per day obtained in our previous study [9] was used to calculate that, with 80% power, we would detect a difference of 1.5 cigarettes smoked per day (which is a size of importance to detect, and consistent with previous research, Lee et al. 2021) as significant at a two‐sided significance level of 5%, with 210 participants available for analysis (105 per sequence group). To account for potential attrition, 252 participants were recruited for randomization.

### Statistical analysis

Intention‐to‐treat analyses of the primary and secondary outcome included all randomized participants who completed at least one intervention period. A mixed‐effects normal regression model was used to estimate the mean difference according to pack size condition, with a 95% confidence interval (CI) and *P*‐value. This involved a repeated‐measures analysis with terms included for the treatment effect, period effect (intervention periods 1 and 2) and order effect [(a–b) or (b–a)]. Evidence for a treatment × order interaction was examined. As pre‐specified in the statistical analysis plan, the following variables were examined as potential analysis model covariates: (1) self‐reported number of cigarettes smoked per day at enrolment; (2) HSI, as measured at enrolment; (3) MTSS, as measured at enrolment; (4) price per cigarette for packs of 20 and packs of 25 cigarettes; (5) duration of ‘usual behaviour’ period; and (6) number of non‐study cigarettes smoked.

## RESULTS

### Participant characteristics

**FIGURE 1:**
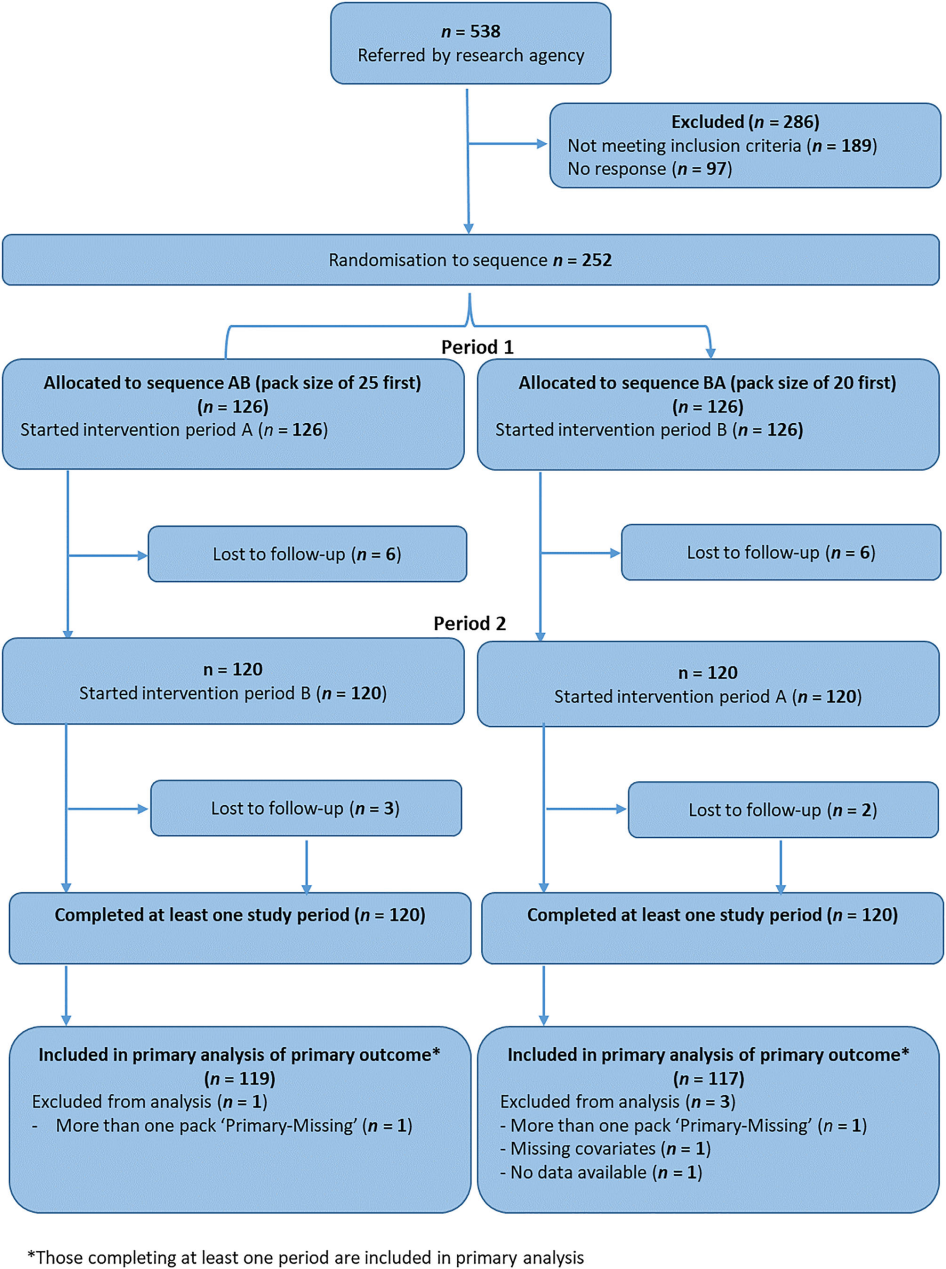
Consolidated Standards of Reporting Trials (CONSORT) flow diagram

Of the 538 smokers referred by the research agency, 349 (65%) met the eligibility criteria, of whom 252 (72%) consented to participate and were randomized to one of the two study arms (Figure 1). Of those randomized, 240 (95%) completed the study; 236 (94%) provided sufficient data for the primary analysis of the primary outcome.

Demographic characteristics appeared comparable between participants who completed the study (*n* = 240, 94%) and those who did not (*n* = 12; 6%) (Table 1). Those completing the study were predominantly female (*n* = 171, 71%) and white (*n* = 208; 87%), with a mean age of 46 years (SD = 12). Forty‐five per cent of participants (*n* = 104) had household incomes less than the average of approximately Can$60 000, with 21% (*n* = 51) having one or more university degrees. Participants were deemed to be adherent to study instructions if at least 90% of the cigarette packs from which they had smoked during each of the two intervention periods were of the correct size, the correct cigarette length and brand variant, and if they did not deviate significantly from study instructions. Of the 4952 photographs of cigarette packs received from 240 participants, seven were of the incorrect pack size. The per‐protocol analysis excluded 21 participants that were deemed non‐adherent. See Supporting information, Material 3 for more information.

**TABLE 1:** Demographic and baseline smoking characteristics of participants.

	Packs of 25 first (a–b)		Packs of 20 first (b–a)		
	Completed (*n* = 120)	Lost to follow‐up (*n* = 6)	Completed (*n* = 120)	Lost to follow‐up (*n* = 6)	Total completed (*n* = 240)
Gender, *n* (%)					
Female	90 (75)	5 (83)	81 (68)	3 (50)	171 (71)
Male	30 (25)	1 (17)	39 (33)	3 (50)	69 (29)
Age, mean (SD)	46.1 (11.7)	50.0 (18.2)	46.1 (12.5)	49.3 (14.9)	46.1 (12.3)
Household income, *n* (%)					
Under $10 000	0 (0)	0 (0)	3 (3)	1 (17)	3 (3)
$10 000–29 999	14 (12)	1 (17)	12 (10)	1 (17)	26 (11)
$30 000–59 999	40 (33)	4 (67)	35 (29)	2 (34)	75 (31)
$60 000–99 999	36 (30)	0 (0)	33 (28)	1 (17)	69 (29)
$100 000 and over	23 (20)	1 (17)	32 (27)	1 (17)	55 (23)
Prefer not to answer	7 (6)	0 (0)	4 (3)	0 (0)	11 (4)
Do not know	0 (0)	0 (0)	1 (1)	0 (0)	1 (0)
Highest education level, *n* (%)					
Grade school/some high school	7 (6)	0 (0)	6 (5)	0 (0)	13 (5)
Completed high school	29 (24)	1 (17)	27 (23)	2 (33)	56 (23)
Trade school/community college	41 (34)	4 (67)	41 (34)	2 (33)	82 (34)
Some university, no degree	13 (11)	1 (17)	25 (21)	2 (33)	38 (16)
Completed university degree	24 (20)	0 (0)	20 (17)	0 (0)	44 (18)
Post‐graduate degree	6 (5)	0 (0)	1 (1)	0 (0)	7 (3)
Ethnicity, *n* (%)					
White	105 (88)	6 (100)	103 (86)	6 (100)	208 (87)
Other/multiracial	15 (13)	0 (0)	17 (15)	0 (0)	32 (13)
Cigarettes smoked a day, mean (SD)	17.4 (6.9)	17.1 (6.7)	16.6 (6.4)	17.3 (6.7)	16.2 (7.1)
HIS,^a^ mean (SD)	3.3 (1.1)	3.3 (0.5)	3.2 (1.1)	3.2 (1.3)	3.2 (1.1)
MTSS,^b^ mean (SD)	2.7 (0.8)	2.7 (0.8)	2.6 (0.8)	2.8 (1.3)	2.6 (0.9)
Cigarette purchasing, *n* (%)					
Individual packs, as needed	34 (28)	3 (50)	27 (23)	1 (17)	61 (25)
Multiple packs of 25s	49 (41)	1 (17)	50 (42)	4 (67)	99 (41)
Carton containing multiple packs of 25s	36 (30)	2 (33)	42 (35)	1 (17)	78 (33)
Other	1 (< 1)	0 (0)	1 (< 1)	0 (0)	2 (< 1)

SD, standard deviation.

^a^ HSI = Heaviness of Smoking Index (range = 0–6);

^b^ MTSS = Motivation to Stop Scale (range = 1–7).

Of the 4952 cigarette packs, 58 had at least one piece of information missing, either from the sticker or information relating to the cigarette type (brand variant, length or size). Of the 236 participants included in the primary analysis of the primary outcome, 12 had missing information imputed for the primary outcome. See the OSF registration for the protocol that was followed for handling missing information (https://osf.io/zby94), which assumes any missing packs were missing at random [17].

The data that form the basis of the results presented here are available from the University of Cambridge Research Data Repository (https://www.data.cam.ac.uk/repository), https://doi.org/10.17863/CAM.88739.

### Primary outcome: number of cigarettes smoked per day

#### Primary analysis

Participants smoked fewer cigarettes per day from packs of 20 cigarettes (*n* = 234, mean = 15.7, SD = 7.1) than when using packs of 25 cigarettes (*n* = 235, mean = 16.9, SD = 7.1) [Table 2]. Plots of the data (Supporting information, Material 4) suggested that although there was some evidence of an order effect for just one pack size (i.e. mean consumption of pack size 20 was slightly lower when packs of 20 were received first), this was too small to obscure the larger overall effect of pack size on consumption.

**TABLE 2:** Number of cigarettes smoked per day and motivation to stop smoking.

	Packs of ≥ 25	Packs of 20	Mean difference^b^ (95% CI) (packs of ≥ 25 minus packs of 20)	*P*	Cohen's *d*
	Mean^a^ (SD)	Mean^a^ (SD)			
Number of cigarettes smoked per day					
Primary analysis^c^	16.9 (7.1)	15.7 (7.1)	−1.3 (−1.7, −0.9)	< 0.001	0.81
Per protocol analysis^d^	17.2 (7.1)	16.0 (7.1)	−1.3 (−1.7, −0.8)	< 0.001	−0.79
Sensitivity analysis 1^1^	16.9 (7.1)	15.6 (7.1)	−1.2 (−1.6, −0.8)	< 0.001	−0.76
Sensitivity analysis 2^2^	16.8 (7.1)	15.6 (7.1)	−1.3 (−1.7, −0.9)	< 0.001	−0.81
Sensitivity analysis 3^3^	16.9 (7.1)	15.7 (7.1)	−1.1 (−1.5, −0.7)	< 0.001	−0.69
Motivation to stop smoking^e^	3.1 (1.3)	3.1 (1.2)	−0.3 (−0.4, −0.1)	0.002	−0.41

CI, confidence interval; SD, standard deviation.

^a^ Unadjusted;

^b^ adjusted for pre‐specified covariates (baseline consumption and heaviness of smoking at enrolment);

^c^ *n* = 236;

^d^ *n* = 215;

^e^ Motivation To Stop Smoking Scale [range = 1 (I do not want to stop smoking) to 7 (I REALLY want to stop smoking)]; *n* = 238.

^1^ Data were analysed with no imputation for missing values (and without any of the assumptions required for this); *n* = 224.

^2^ Data were analysed as per the primary analysis, but missing values imputed for up to two cigarette packs classed as Primary‐missing; *n* = 237.

^3^ Data were analysed as per the primary analysis, with an additional covariate: self‐reported mitigating factors impacting on cigarette consumption; *n* = 235.

After adjusting for pre‐specified covariates (baseline consumption and heaviness of smoking at enrolment) modelling (*n* = 236) estimated that participants smoked 1.3 fewer cigarettes per day (95% CI = −1.7 to −0.9) or 7.6% fewer (95% CI = −10.1% to −5.2%) from packs of 20 cigarettes (Table 3). The interaction between pack size and allocation order was investigated but was not retained in the final model (*P* = 0.286). These findings were robust to different approaches to imputation for data missing from 12 participants.

**TABLE 3:** Primary (*n* = 236) and secondary (*n* = 238) outcome model estimates.

	CPD^a^			MTSS^b^		
	Estimate (SE)	95% CI	*P*‐value	Estimate (SE)	95% CI	*P*‐value
Intercept	0.91 (0.95)	−0.96, 2.77	0.344	3.32 (0.26)	2.81, 3.84	< 0.001
Pack size 20 (Ref: pack size 25)	−1.29 (0.21)	−1.70, −0.88	< 0.001	0.21 (0.08)	0.05, 0.37	0.013
Order 20 first (Ref: 25 first)	0.40 (0.55)	−0.66, 1.47	0.460	−0.18 (0.16)	−0.49, 0.14	0.276
Period 2 (Ref: period 1)	0.23 (0.21)	−0.19, 0.64	0.286	–	–	–
CPD^a^ baseline	0.74 (0.06)	0.63, 0.85	< 0.001	−0.01 (0.02)	−0.04, 0.02	0.593
HSI^c^	0.93 (0.34)	0.27, 1.58	0.007	0.01 (0.09)	−0.17, 0.19	0.913
Pack (20) by order (20 first)	–	–	–	−0.46 (0.12)	−0.69, −0.23	< 0.001

When including the interaction term, cannot include the period otherwise terms are aliased.

CI, confidence interval.

^a^ CPD = cigarettes smoked per day;

^b^ MTSS = motivation to stop scale (range = 1–7);

^c^ HSI = heaviness of smoking index (range = 0–6); SE = standard error.

#### Sensitivity analyses

Four planned sensitivity analyses of the primary outcome were conducted (Table 2). The results of each of these was compatible with the primary analysis. See Supporting information, Table S3.1 for full details of these models.

In an analysis with no imputation (*n* = 224), after adjusting for pre‐specified covariates, modelling estimated that participants smoked 1.2 fewer cigarettes per day (95% CI = −1.6 to −0.8), or 7.3% fewer (95% CI = −9.8 to −4.7%) from packs of 20 cigarettes.

Similarly, in an analysis in which up to two packs with missing data would be imputed (*n* = 237), modelling estimated that participants smoked 1.3 fewer cigarettes per day (95% CI = −1.7 to −0.9) or 7.7% fewer (95% CI = −10.1 to −5.2%) from packs of 20 cigarettes.

In a per‐protocol analysis (*n* = 215), modelling estimated that participants smoked 1.3 fewer cigarettes per day (95% CI = −1.7 to −0.8) or 7.4% fewer (95% CI = −10.0 to −4.9%) from packs of 20 cigarettes.

Finally, in an analysis similar to the primary analysis but with the addition of a variable for self‐ reported mitigating factors such as illness (*n* = 235), modelling estimated that participants smoked 1.1 fewer cigarettes per day (95% CI = −1.5 to −0.7) or 6.5% fewer (95% CI = −8.9 to −4.1%) from packs of 20 cigarettes.

### Secondary outcome: motivation to stop smoking

Randomization balanced the baseline motivation to stop smoking between the study arms. The MTSS scores of participants allocated to both sequence orders [(a–b) and (b–a)] increased from baseline to the end of the first intervention period (mean = 0.2, SE = 0.1, *P* = 0.021). Participants randomized to smoke from packs of 25 cigarettes first had greater increases in MTSS scores from baseline compared to those allocated to smoke first from packs of 20 (mean = 0.3, SE = 0.1, *P* = 0.009). When modelling MTSS recorded during the study (*n* = 238, Tables 2 and 3), the interaction between pack size and allocation order was investigated and retained in the model (*P* < 0.001). This showed that MTSS tended to increase from intervention period 1 to intervention period 2, regardless of the pack sizes received.

### Other outcomes

Responses to the end of study questionnaire are described in Supporting information, Material 6. We report here only responses to the question regarding preferred pack size, following completion of the study.

Eighty‐two per cent of participants (*n* = 234) preferred using packs of 25, 16% preferred using packs of 20 and 2% expressed no preference. For those preferring pack sizes of 25, the most cited reason was that the packs of 25s lasted longer (*n* = 62). Among those preferring pack sizes of 20, the most cited reason was that they smoked fewer cigarettes (*n* = 15) (see Box 1).

Box 1 **Preferences for pack sizes of 25 and 20.**

*Prefer 25s (82%)*

Packs of 25 last longer

‘I prefer packs of 25 because I can usually make 2 packs last for 3 days, but with the smaller size pack 2 packs were not enough for 3 days’

Packs of 25 are better value

‘I preferred the pack of 25 as it is better value for the money’

Packs of 25 contain more cigarettes

‘My preference is to buy packs of 25 simply because there are more cigarettes per pack’

*Prefer 20s (16%)*

Reduces smoking

‘Definitely prefer 20s. I didn't ‘suffer’ at all scaling down from my years‐long smoking of 25 cigarettes to 20 cigarettes per day’

‘I prefer the 20 packs now, because I do smoke less’

Packs of 20 are cheaper

‘Seemed cheaper buying 20s because there was often a discount for buying multiple packs’

Smaller physical size of packs of 20

‘I liked the 20 because the pack is smaller and fit better in my pocket’

## DISCUSSION

Using packs of 20 compared with 25 cigarettes reduced the mean number of cigarettes smoked per day by 1.3, a reduction of 7.6%. Motivation to stop smoking was similar when smoking from either pack size. This study therefore provides the strongest evidence to date that smaller pack sizes reduce the number of cigarettes smoked. This finding was robust to four sets of sensitivity analyses.

### Comparison with previous findings

The results of the current study provide the first evidence that at least part of the observed association between cigarette pack size and consumption—those smoking from larger pack sizes smoke more cigarettes [11]—is causal. These results also fitted with the more robust evidence from studies of food and alcohol consumption showing that smaller portion and pack sizes reduce consumption [10, 18]. They are also consistent with the growing evidence from studies of alcohol consumption showing similar effects on consumption of reducing glass and bottle sizes [19, 20]. While food, alcohol and tobacco vary in the extent to which they are addictive, consumption of even the most addictive—tobacco—is influenced nonetheless by external cues such as constraints on where it can be consumed, with some evidence that such constraints lead to voluntary restrictions on home smoking [21].

### Interpretation of findings

A reduction in cigarettes smoked is an appropriate aim for tobacco control policy if it leads either to direct or indirect health benefits. Therefore, one critical question is whether the magnitude of reduction observed in this study is likely to translate to population health benefit. A decrease of 1.3 cigarettes per day, or fewer than 10% of usual cigarettes smoked, is unlikely to confer any direct reduction in risk due to decreased exposure. First, smoking reduction studies suggest that a minimum reduction of 50% consumption is required to reduce indicators of risk [22, 23]. Secondly, smokers typically engage in compensatory smoking, whereby reductions in cigarette consumption at the individual level are offset by compensatory increases in smoking behaviour, such as taking more puffs and deeper inhalation, in an effort to maintain nicotine intake [24]. Compensatory changes to reductions in cigarettes per day have also been observed at the population level: average cigarette consumption has declined in the United States over several years, but nicotine intake among smokers has remained unchanged [25]. Rather, the extent to which a reduction in cigarettes per day might contribute to harm reduction depends, in part, on the extent to which smoking fewer cigarettes a day increases the chances of quitting smoking, indirectly leading to health improvement.

There is evidence that smoking fewer cigarettes per day—which may be a marker of lower dependence—increases the probability of cessation. Smoking one fewer cigarette per day at baseline was estimated in one study to increase the chances of being a former smoker by between approximately 7 and 11% [26]. There is also evidence that deliberate attempts to reduce cigarette consumption increase the likelihood of subsequent cessation [27]. However, the impact of interventions that aim to reduce cigarette consumption that do not require deliberate effort on the part of the cigarette smoker is unknown. It is possible that reduced consumption might, over time, lead to extinction of conditioned associations between the behaviour of smoking (and associated cues) and the reward association with nicotine consumption [28, 29]. However, this may not occur for an average reduction of one cigarette per day and, if it does, it is unknown whether it would be of sufficient magnitude to reduce dependence and promote cessation. The current study was not designed to address these questions. Therefore, while we provide evidence that capping pack size might lead to a reduction in cigarette consumption—an important proof of concept—the impact on population smoking rates remains unknown. We judge the potential effect likely to be small or negligible.

Motivation to stop smoking was low in this sample of smokers—that is, below the mid‐point of a scale for motivation to stop smoking—reflecting the inclusion criterion that participants did not intend to quit smoking in the next 3 months. An unpredicted interaction between pack size and order was found, such that motivation to stop smoking increased from intervention period 1 to intervention period 2 regardless of which pack size was used first, and participants who were allocated to use packs of 20 cigarettes first had lower motivation to stop smoking at the end of both intervention periods compared to participants who were allocated to use packs of 25 cigarettes first.

### Strengths

This is the first experimental study, to our knowledge, to estimate the impact on daily cigarette consumption of smoking from smaller packs. Retention of randomized participants was very high which, together with the study design and procedures, minimized the risk of bias.

### Limitations

The two main limitations of the study concern: first, its generalizability to other populations, settings and pack sizes; and secondly, the lack of biochemical measures of nicotine exposure. The study was conducted in Canada where, in the majority of provinces, cigarettes are sold in only two pack sizes—20 and 25. This was an advantage for the current study. There is no reason why smokers in Canada should differ from those elsewhere, but this remains an uncertainty. Participants were broadly representative of smokers in Canada in terms of their age, income and level of education. They were less representative in terms of gender—with women being over‐represented—and in terms of ethnicity—with white groups over‐represented. While there are no a priori reasons why these differences should affect the impact of the intervention, this merits some caution. The further limitation of the study was the absence of biochemical measures of nicotine to assess the extent to which any reduction in cigarettes per day was associated with a reduction in nicotine exposure. While this would have provided some evidence of the extent to which reducing cigarettes per day resulted in compensatory smoking, this was beyond the scope of the current study.

### Implications for research

The current study raises several questions for further research. These concern first, the mechanisms for the observed effect and secondly, the optimal pack size for cigarettes. Understanding the mechanisms by which cigarette pack size affects consumption could provide the basis for optimizing the intervention [30]. Free text responses to the end of study questionnaire were compatible with two linked possible explanations for what is known as ‘the portion size effect’—the tendency to consume more the larger the portion or package [31]. First, smaller packs might reduce consumption by making it more effortful to smoke more—that is, to buy or open a new pack [10]. Secondly, it might reflect the tendency to consume a specific number of units in a pre‐specified period of time [32]. This might be one glass or bottle of wine with dinner, and one pack of cigarettes in a day or during a 2‐day period. When the glass, bottle or pack contain more, more is consumed. Regarding the optimal cigarette pack size, the current study compared just two pack sizes. It is unknown whether the size of effect observed is linear—that is, whether smoking from a pack size of 25 compared with a pack of 30 would reduce the number of cigarettes smoked daily by the same proportion—approximately 7%—as that observed in the current study when smoking from a pack size of 20 compared with a pack of 25.

### Implications for policy

The novel findings from this study raise questions regarding whether pack size might be a useful target for intervention in addition to existing tobacco control policies.

There are at least two ways to shift smokers away from buying larger packs, which are not mutually exclusive. The first is to use price‐based measures to ensure that purchasing cigarettes and tobacco in larger quantities—in packs or bundles of packs in cartons—does not provide value for money. As noted by some participants in the current study (Box 1), their preferences for larger packs reflected better value for money; that is, paying less per cigarette when buying in packs of 25 than in packs of 20.

Proportionate pricing of cigarettes and hand‐rolling tobacco is needed so that the price per stick or gram of tobacco is the same, regardless of pack size. This builds upon the robust evidence that tax and price increases are the most impactful tobacco control policies, particularly for children and young adults [33]. The second approach, to complement such price‐based measures, is to regulate the maximum size at which cigarettes and tobacco can be sold. An increasing number of countries have regulated a minimum pack size of 20 to reduce the affordability of cigarettes to children [3] resulting, for example, in a virtual disappearance of pack sizes below 20 in the European Union [6]. A few have regulated to cap the maximum size at 20 [3]. Different tobacco control policies may therefore need to be balanced to find an optimum pack size. While the current study cannot be used to determine whether and at what size a maximum cap might be set, it provides the first experimental evidence that people smoke more when smoking from larger pack sizes.

### CONCLUSION

Smoking from packs of 20 compared with 25 cigarettes reduced the number of cigarettes smoked per day. The extent to which the observed reduction would translate into reduced population rates of smoking remains uncertain.

## DECLARATION OF INTERESTS

None.

## TRIAL REGISTRATION

ISRCTN: 16013277 https://www.isrctn.com/ISRCTN16013277.

## AUTHOR CONTRIBUTIONS

Theresa M. Marteau conceptualised the study with methods input from Gareth J. Hollands, Marcus R. Munafò, David Hammond. Ilse Lee administered the study and collected and cleaned data together with Anna K. M. Blackwell, Alice Hobson, Danielle Wiggers. Mark A. Pilling and Katie De‐loyde curated the data and conducted the statistical analyses. Ilse Lee, Theresa M. Marteau, Mark A. Pilling drafted the manuscript with all authors providing critical revisions. Theresa M. Marteau is guarantor.

## Supporting information


**Data S1:** Instructions for participants
**Data S2:** Survey questions
**Data S3:** Missing data and adherence
**Data S4:** Order effect
**Data S5:** Sensitivity analyses
**Data S6:** Survey results

## Footnotes

1 British Columbia was excluded as cigarettes are only sold there in packs of 20 cigarettes. The Territories were excluded as the research agency did not have research panels there.

2 One participant was allowed to shorten this to 6 days to avoid a clash with scheduled surgery.
