# Supplementary material for: Cigarette pack size and consumption: a randomized cross‐over trial
Source: Addiction. 2022 Nov 3;118(3):489–99. doi: 10.1111/add.16062 (PMC10100265; doi:10.1111/add.16062)
Supplement: Supplementary file 1 — Data S1: Instructions for participants Data S2: Survey questions Data S3: Missing data and adherence Data S4: Order effect Data S5: Sensitivity analyses Data S6: Survey results [file ADD-118-489-s001.docx]

## Supplementary Material

### Contents

Supplementary material 1: Instructions for participants

Supplementary material 2: Survey questions

Supplementary material 3: Missing data and adherence

Supplementary material 4: Order effect

Supplementary material 5: Sensitivity analyses

Supplementary material 6: Survey results

## Supplementary Material 1: Instructions for participants


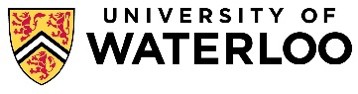

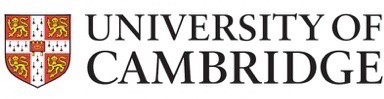


**Cigarette Packs and Health Warnings: Instructions for participants**

**Study Overview**

**
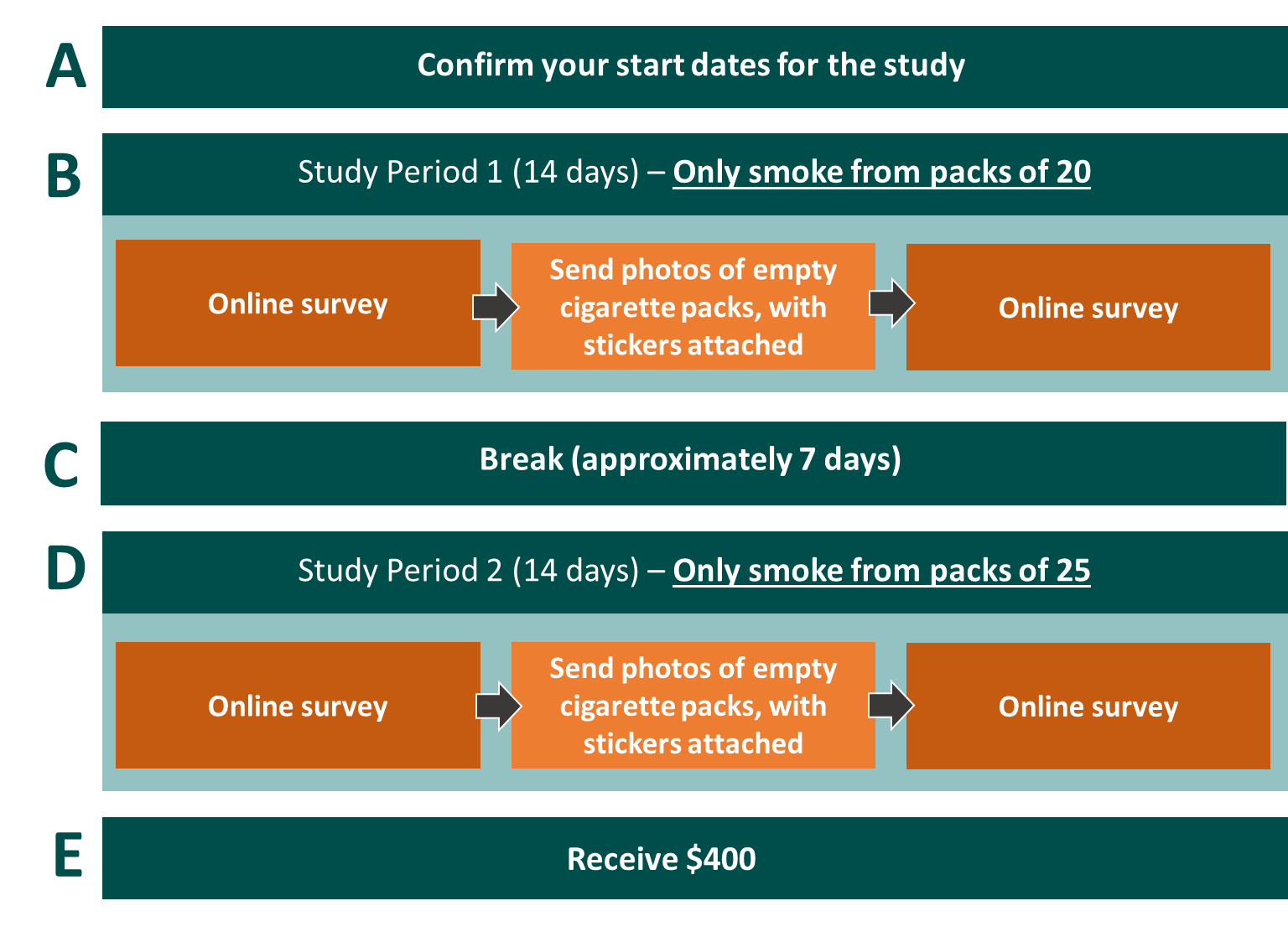
**

The diagram above gives an overview of the study. Read on for further instructions.

If you do not do follow the instructions, you may be excluded from the study so please read them carefully and contact us if you have any questions.

**Contents**

**A.** Confirm your start dates ……………………………………….. 2

**B.** Study Period 1 ……………………………………………………… 2

**C.** Break …………………………………………………………………….. 2

**D.** Study Period 2 …………………………………………………….. 3

**E.** Receive $400 ………………………………………………………. 3

FAQs:

What if I cannot find the right cigarettes ……………………. 3

What happens if I do not meet a deadline? ……………….. 3

How should I fill in the stickers? …………………………….….. 4

How should I attach and photograph the stickers? ……. 5

**Contact details** …………………………………………………………… 6

**The Study in Detail**


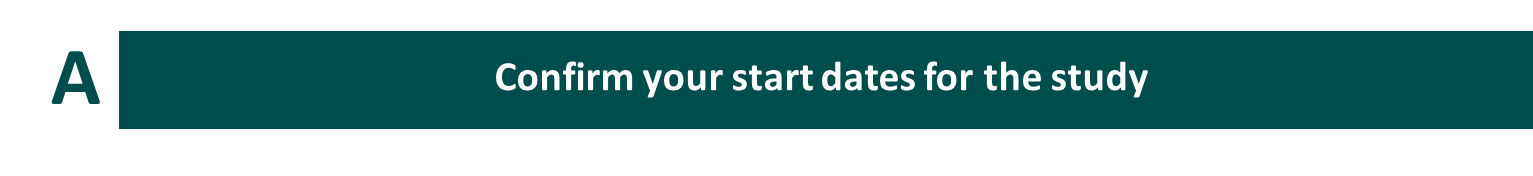


1. Respond ‘yes’ to our email asking you to confirm your start date for Study Period 1.
2. Please place the pack of 25 cigarettes that you bought at the start of the study in the envelope labelled ‘Save for Study Period 2’. This is to remind you not to smoke this pack during Study Period 1, and to save it for Study Period 2.


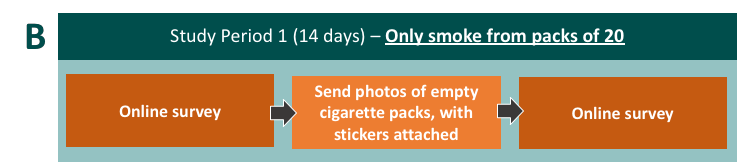


1. From your start date, only smoke «cig_brand_and_length» cigarettes from pack sizes of 20.

**Important:** We want to ensure that the health warnings you see are the correct size for each study period; therefore, wherever possible you should smoke only from your own cigarette packs. We understand that there may be times when you smoke from another pack (e.g. being given a cigarette by a friend or family member) and these cigarettes should be recorded on stickers.

1. If you are partway through a cigarette pack at the start of Study Period 1, please save it until after the study period.
2. Complete the one-question survey that will be sent to you on day 1.
3. Attach a completed sticker to the front of each pack. See page 4 for more information about this.
4. On day 7, send us photos of every pack you finished between days 1 and 7. On day 14, wait until you have smoked your last cigarette of the day, then send us photos of every pack you finished between days 8 and 14, and any packs you are partway through smoking. See page 5 for more information on this step.
5. On day 14, you will receive a short online survey to complete.


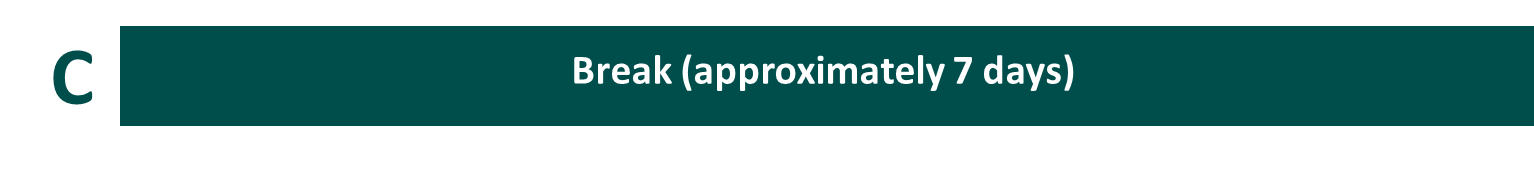


1. There will be approximately one week between the end of Study Period 1 and the start of Study Period 2. During this time you can smoke cigarettes from packs of any size.


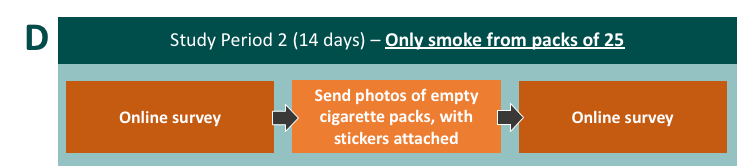


1. From your start date, only smoke «cig_brand_and_length» cigarettes from pack sizes of 25.
2. Apart from using a different size of cigarette pack, the steps involved in Study Period 2 are identical to Study Period 1.

**
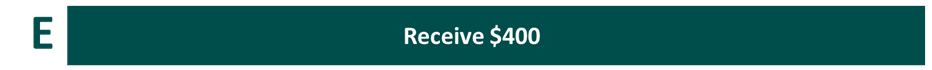
**

If you have completed all aspects of the study, you will receive $400. The payment will be made by Leger via virtual visa gift card within 7 weeks of completing the study.

**Frequently Asked Questions**

**What if I cannot find the right cigarettes?**

If for some reason, you cannot find your usual brand variant of cigarettes in the correct pack size, please contact us as soon as possible and we will discuss the best course of action with you.

While we discuss what to do next, please try to use a cigarette pack of the same size you were asked to use in that study period. Please keep hold of any alternative cigarette packs you have had to use. Complete a sticker for them and send us photos of them just as you would have for your normal packs.

**What happens if I do not meet a deadline?**

We will ask you to complete each step of this study by a certain date, usually within two or three days.

Some deadlines cannot be changed, and we will not be able to continue to include you in the study if you do not meet them. Some deadlines are more flexible, and we will signal this in our emails by asking you to contact us if you need more time.

**How should I fill in the stickers?**

1. Fill out a sticker as soon as you finish a pack, rather than trying to remember the information later.
2. Keep a note of any cigarettes that:
   1. You give to other people
   2. You smoke from other packs (e.g. given to you by a friend).
3. Use a pen to fill in the stickers to avoid your answers rubbing off.

Participant ID. This is in every email we send you.

Fill in the date you finished the pack, writing the month as a word, not a number.

*E.g. 3 March 20, not 3/3/20.*


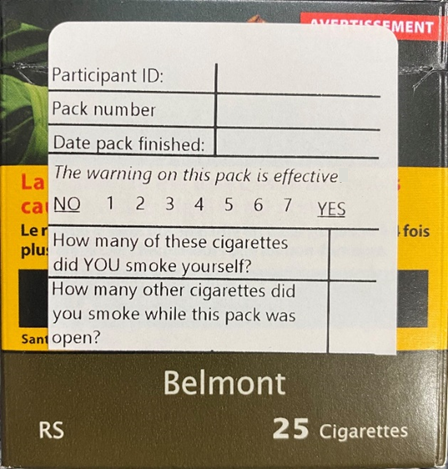


Number your packs in the order you smoke them. *E.g. the first pack you smoke = 1, the second = 2.*

Start again from ‘1’ at the beginning of Study Period 2.

Fill in the number of cigarettes you smoked from this pack.

*E.g. if you gave two cigarettes from this pack of 25 to a friend and smoked the rest, write 23.*

How much do you agree with this statement?

Circle a number from 1 (Not at all) to 7 (Extremely)

Fill in the number of other cigarettes you smoked, that were not from this pack or from any of your other packs, while this pack was open.

*E.g. cigarettes given to you by a friend.*

1. If you are partway through a pack of cigarettes on day 14**,** please complete the “Last day of study period” sticker, and attach it to the front of the pack, along with the usual sticker.
2. Do not cover any information on the usual sticker, the brand name, or the pack size.
3. Once you have sent us a photograph of the pack, you can remove both stickers, and continue to smoke from the pack as normal.


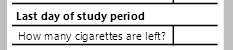


Fill in the number of cigarettes left in the pack

**How should I attach and photograph the stickers?**

1. Take a photo of each individual pack of cigarettes.
2. Make sure you have attached a sticker to the front of the pack.
3. Make sure that the whole cigarette pack is visible, including the brand name and pack size.
4. Make sure that the photo is not blurry.

**
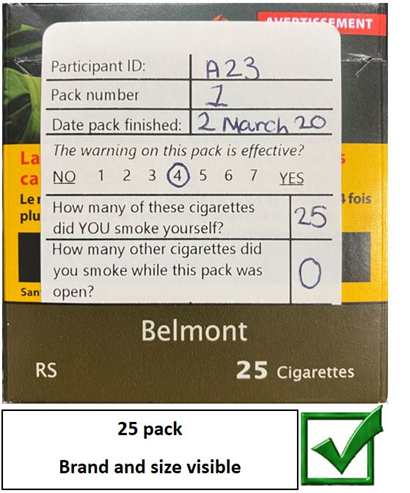
**

**
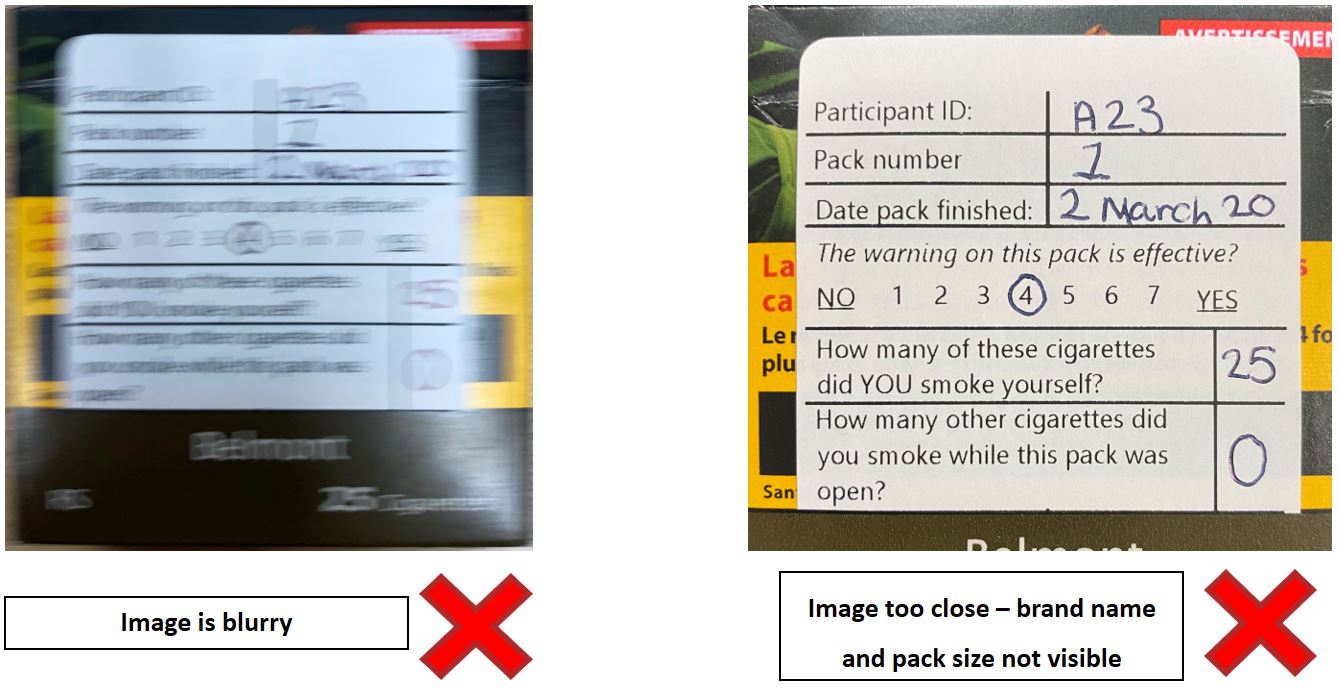
**

**What if the brand information is on a different side of the pack to the pack size?**

If it is not possible to show the brand, pack size and sticker in the same photo (e.g. if the pack size is displayed on the opposite side of the pack to the brand information) please do one of the following:

**Option 1 (for finished packs only)**

Unfold and flatten out the empty cigarette packs so that all sides of the packaging can be seen in the photo. For any packs that you are only partway through, use option 2 or 3 below.


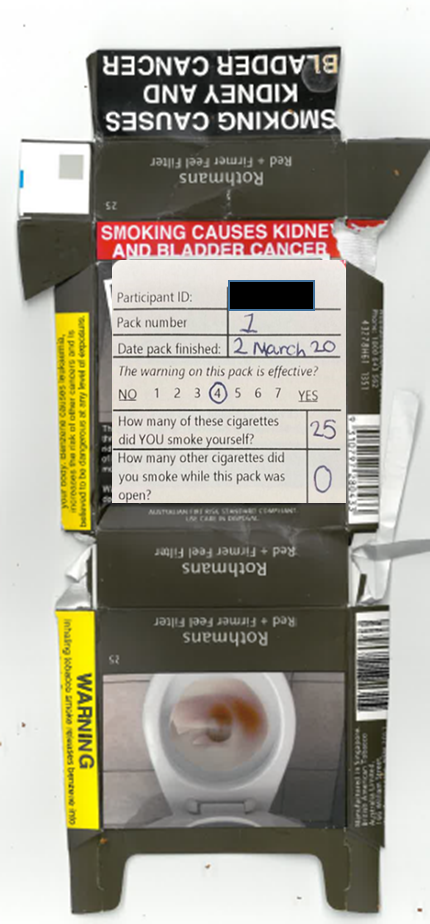


**Option 2**

Take two photos of each pack – one from the front and one from the back –to allow all of the information to be captured. If you can, make it clear which photos belong together (e.g. by writing the pack number on the side of the pack which does not have the sticker).

**Option 3**

Capture all of the fronts of the cigarette packs in one photo and all of the backs of the cigarette packs in another photo. Please keep the packs in the same order for each photo.

**Contact Details**

If you have any questions about these instructions, or are unclear about anything else in the study, please do not hesitate to get in touch with the research team via email ([smokingstudy@uwaterloo.ca](mailto:smokingstudy@uwaterloo.ca)).

**Supplementary Material 2: Survey Questions**

**End of Intervention Period survey**

At the end of each of the two intervention periods, the following questions will be asked to each participant using an online survey:

- 1. How effective did you find the warning labels on your cigarette packs over the last two weeks? (Rating from 1 (Not at all) – 7 (Extremely)
  2. How many cigarettes do you think you smoked in the last two weeks compared to the number you usually smoke?
     1. Fewer
     2. About the same
     3. More
     4. Don’t know
  3. In the last 2 weeks, have you changed how much smoke you inhale from each cigarette, by changing the number of puffs or how deeply you inhale?
     1. Less smoke than usual
     2. About the same
     3. More smoke than usual
     4. Don’t know
  4. Is there anything that affected your usual pattern of smoking over the last two weeks?
  5. Did Covid-19 affect the number of cigarettes you bought in the last two weeks?
     1. No
     2. Yes – I bought more cigarettes in the last two weeks because of Covid-19.
     3. Yes – I bought fewer cigarettes in the last two weeks because of Covid-19.
  6. Did Covid-19 affect the number of cigarettes you smoked in the past two weeks?
     1. No
     2. Yes – I smoked more cigarettes in the last two weeks because of Covid-19.
     3. Yes – I smoked fewer cigarettes in the last two weeks because of Covid-19.

## End of Study survey

At the end of the study (the end of Intervention Period 2), the following questions will be asked to each participant using an online survey:

1. Do you try to make your usual pack of 25 cigarettes last a certain amount of time?
   1. Always
   2. Sometimes
   3. Never

If you answered (a) or (b), please answer Questions 2 and 3 below:

1. How long is this? …..days or …….hours
2. Did you try to keep to the same amount of time for pack sizes of 20?
   1. Yes
   2. No

Thinking about the two weeks when you were smoking from packs of **20 cigarettes**, please answer the following questions…

1. When you finished a pack did you usually:
   1. have another pack ready to start
   2. go out to buy another pack

If you answered (b), please answer question 5 below.

1. When did you usually go out to buy another pack:
   1. Immediately, or as soon as I realistically could e.g. first thing in the morning if I finished the pack at night
   2. I waited

If you answered (b), how long did you try to wait? hours

1. How did you buy your cigarettes when you were buying them in packs of 20?
   1. in individual packs as I needed them
   2. in multiple packs of 20s
   3. in a carton containing multiple packs of 20 cigarettes
   4. other (please specify)
2. How often did you buy cigarettes when you were smoking from pack sizes of 20 compared to packs of 25?
   1. Less often
   2. More often
   3. The same

Thinking about the two weeks when you were smoking from packs of **25 cigarettes**, please answer the following questions…

1. When you finished a pack did you usually:
   1. have another pack ready to start
   2. go out to buy another pack

If you answered (b), please answer question 9 below.

1. When did you usually go out to buy another pack:
   1. Immediately, or as soon as I realistically could e.g. first thing in the morning if I finished the pack at night
   2. I waited

If you answered (b), how long did you try to wait? hours

1. How did you buy your cigarettes when you were buying them in packs of 25?
   1. in individual packs as I needed them
   2. in multiple packs of 25
   3. in a carton containing multiple packs of 25 cigarettes
   4. other (please specify)
2. Which is better value for money for you:
   1. Buying cigarettes in pack sizes of 20
   2. Buying cigarettes in pack sizes of 25
   3. No difference
   4. Don’t know
3. Thinking about your experiences of the two cigarette pack sizes (20 and 25), please indicate in the box below which size you preferred, and why.
4. Why do you normally purchase cigarettes in packs of 25?
5. Thinking again about your experiences with each cigarette pack size, please indicate in the box below which size you would prefer to buy from now and why.
6. Please use the space below to briefly tell us what you think the study was about
7. Thinking about your experiences with each cigarette pack size, do you think the different pack sizes had any impact on your smoking or how much you smoked, and if so why?
8. Please use the space below to make any other comments about this study.
9. [After participants have been debriefed on the study purpose] Now you know what the study was about, are there any comments you would like to make?

**Supplementary Material 3: Missing data and adherence to instructions**

**Missing data**

In total, we received photos of 4,952 cigarette packs. Table 1 presents the number of cigarette packs that were returned with any information relating to the cigarette brand variant, length or size or information on the sticker missing. The total number of cigarette packs that had any information missing was 58.

Table 1. Number of cigarette packs returned with information missing.

| **Cigarette pack variables** | **Number of packs returned with this information missing n = 4,952 (%)** |
| --- | --- |
| Cigarette brand | 5 (0.1) |
| Cigarette brand variant | 21 (0.4) |
| Cigarette length | 10 (0.2) |
| Pack size | 4 (0.1) |
| Number of cigarettes smoked from this pack | 5 (0.1) |
| Number of other cigarettes smoked | 24 (0.5) |
| Number of cigarettes remaining (partially empty packs only)  n = 279 | 2 (0.7) |
| Date finished | 4 (0.1) |

**Adherence to instructions**

**Adherence to cigarette pack brand variant, length and pack size**

Of the 4,952 photos of cigarette packs returned during the study, 115 were not of the correct pack size, brand variant and cigarette length. Table 2 presents the number of cigarette packs that were non-adherent to each criterion.

Table 2. Adherence to cigarette brand, pack size, cigarette length and variant

|  | **Number of cigarette packs (%)** |
| --- | --- |
| Incorrect pack size | 7 (0.1) |
| Incorrect brand variant | 88 (1.8) |
| Incorrect cigarette length | 26 (0.5) |
| Incorrect pack size, variant or length | 115 (2.3) |

**Participants excluded for non-adherence**

A total of 21 participant were excluded from the per-protocol analysis for not being adherent to study instructions. Table 3 shows the number of participants excluded for each reason.

Table 3.

| **Reason for non-adherence** | **Number of participants** |
| --- | --- |
| Less than 90% of their packs were of the correct cigarette brand variant, cigarette length or pack size | 18 |
| Significant deviation from study instructions | 3 |
| Participant said they split the packs with their partner in the final survey. | 1 |
| Participant said that they had misunderstood the study instructions in the final survey. They thought that they were only supposed to use one pack in each week of the study period, rather than smoke as usual. | 1 |
| Participant filled in extra numbers on their stickers and it was unclear whether they had recorded the correct information. | 1 |

**Supplementary Material 4: Order effect**

The figure shows some evidence of an order effect for just one pack size (i.e. mean consumption of pack size 20 was slightly lower when packs of 20 were received first), this was too small to obscure the larger overall effect of pack size on consumption.


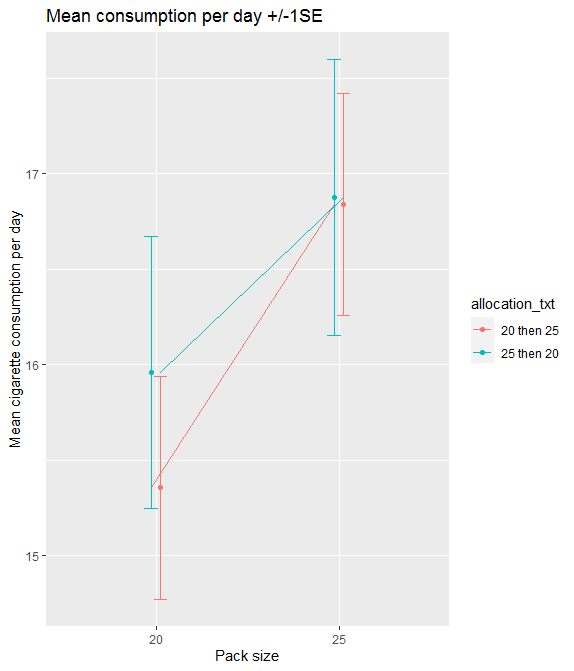


**Supplementary Material 5: Sensitivity analyses**

**Table S3.1. Sensitivity analyses of cigarettes per day**

|  | Per protocol (n=215) |  |  | Analysis1: No imputation  (n=224) |  |  |
| --- | --- | --- | --- | --- | --- | --- |
|  | Estimate (St.Error) | 95%CI | P-value | Estimate (St.Error) | 95%CI | P-value |
| Intercept | 1.18  (1.01) | -0.78,  3.15 | 0.242 | 0.36  (0.98) | -1.55,  2.27 | 0.715 |
| Pack size 20  (Ref: 25) | -1.28  (0.23) | -1.72,  -0.84 | <0.001 | -1.21  (0.22) | -1.64,  -0.79 | <0.001 |
| Order 20 first  (Ref: 25 first) | 0.39  (0.57) | -0.72,  1.51 | 0.490 | 0.49  (0.56) | -0.61,  1.59 | 0.384 |
| Period 2 (Ref: 1) | 0.18  (0.22) | -0.26,  0.62 | 0.422 | 0.25  (0.22) | -0.17,  0.67 | 0.251 |
| CPDa baseline | 0.74  (0.06) | 0.62,  0.85 | <0.001 | 0.79  (0.06) | 0.67,  0.91 | <0.001 |
| HSIb | 0.93  (0.35) | 0.24,  1.61 | 0.009 | 0.82  (0.35) | 0.15,  1.50 | 0.018 |

*aCPD = Cigarattes smoked Per Day*

*bHSI = Heaviness of Smoking Index (range 0 - 6)*

|  | Analysis2: Imputation2 (n=237) |  |  | Analysis3: Mitigating factor (n=235) |  |  |
| --- | --- | --- | --- | --- | --- | --- |
|  | Estimate  (St.Error) | 95%CI | P-value | Estimate  (St.Error) | 95%CI | P-value |
| Intercept | 0.88  (0.95) | -0.97,  2.73 | 0.354 | 1.00  (0.91) | -0.78,  2.77 | 0.275 |
| Pack size 20  (Ref: 25) | -1.29  (0.21) | -1.70,  -0.88 | <0.001 | -1.09  (0.21) | -1.50,  -0.69 | <0.001 |
| Order 20 first  (Ref: 25 first) | 0.40  (0.55) | -0.67,  1.46 | 0.467 | 0.26  (0.52) | -0.76,  1.29 | 0.614 |
| Period 2 (Ref: 1) | 0.23  (0.21) | -0.18,  0.64 | 0.275 | 0.05  (0.21) | -0.36,  0.45 | 0.825 |
| CPDa baseline | 0.74  (0.06) | 0.63,  0.85 | <0.001 | 0.74  (0.06) | 0.63,  0.85 | <0.001 |
| HSIb | 0.94  (0.33) | 0.28,  1.59 | 0.006 | 0.88  (0.32) | 0.25,  1.51 | 0.007 |
| Mitigating circumstancesc | - | - | - | 0.19  (0.28) | 0.04,  2.14 | <0.001 |

*aCPD = Cigarettes smoked Per Day*

*bHSI = Heaviness of Smoking Index (range 0 - 6)*

*cMitigating circumstances during a period (-1 causing a reduction, 0 no change, +1 causing an increase)*

## Supplementary Material 6: Survey results

**Cigarette supply**

At the beginning of each intervention period, participants were sent a one-question survey to ask how many cigarette packs they had at home of the correct size for the intervention period. See Table 1 for a summary of these results in terms of the number of packs and the number of cigarettes.

### Table S4.1. Shows a) the number of cigarette packs of the correct size that participants reported having at home at the start of each intervention period and b) the number of cigarettes this equates to.

**Intervention period**

**Packs of 20**

(n = 234)

### Packs of 25

(n = 234)

### Number of cigarette packs of the correct size.

- 1. **Number of cigarettes contained in cigarette packs of the correct size.**

4.00 (4.78) 4.32 (5.48)

80.17 (95.64) 107.96 (136.95)

## End of Intervention Period survey

At the end of each intervention period, participants completed the End of Intervention Period survey. Please see Table 2 for a summary of the results.

### Table 2. Participant responses to the End of Intervention Period survey, separated according to intervention period (Packs of 20 or Packs of 25).

|  | **Intervention period** | |
| --- | --- | --- |
|  | **Packs of 20 n (%)**  (n = 232) | **Packs of 25 n (%)**  (n = 234) |
| In the last 2 weeks, have you changed how much smoke you inhale from each cigarette, by changing the number of puffs or how deeply you inhale?   1. Less smoke than usual 2. About the same 3. More smoke than usual 4. Don’t know Missing | 35 (15)  162 (70)  26 (11)  8 (3)  1 (0) | 10 (4)  207 (88)  6 (3)  11(5)  0 (0) |
| Did Covid-19 affect the number of cigarettes you bought in the last two weeks? |  |  |

| a. No | 197 (85) | 187 (80) |
| --- | --- | --- |
| b. Yes – I bought more cigarettes in the last two | 26 (11) | 39 (17) |
| weeks because of Covid-19. |  |  |
| c. Yes – I bought fewer cigarettes in the last two |  |  |
| weeks because of Covid-19. | 8 (3) | 8 (3) |
| Missing | 1(0) | 0(0) |
| Did Covid-19 affect the number of cigarettes you smoked in the past two weeks?   1. No 2. Yes – I smoked more cigarettes in the last two weeks because of Covid-19. 3. Yes – I smoked fewer cigarettes in the last two weeks because of Covid-19.   Missing | 194 (84) | 189 (81) |
|  | 29 (13) | 37 (16) |
|  | 8 (3) | 8 (3) |
|  | 1 (0) | 0 (0) |

**End of Study survey**

Analysis of the End of Study survey (n = 234) indicated that a quarter of participants said they always make their usual pack of 25 cigarettes last a certain amount of time and just over half (53%) said they sometimes do. Of the 181 participants who said they make their packs of cigarettes last a certain length of time, the mean length of time was 47.5 hours (range: 4-336 hours, SD = 29.2), with the most commonly reported length of time being 48 hours (n=72), followed by 24 (n =34) and 72 (n=22). Of the 22 participants who answered the question of whether they kept to the same amount of time when using packs of 20 during the study, 82% said that they did. When participants were smoking from packs of 25 cigarettes, 85% said they had another pack ready to be started when they finished a pack and of those who did not have a pack ready to go, 91% said they immediately went to the shops to buy one. These results were very similar when participants were smoking from packs of 20s. Participants reported a very similar pattern of purchasing behaviour when buying packs in the two pack sizes with roughly 20% buying them individual packs as they needed them, half buying multiple packs at a time and a third buying cigarettes in cartons. Nearly half of participants (49%) reported that they purchased cigarettes as often when they were using packs of 20s as when they were using packs of 25s, 41% reported purchasing them more often when using packs of 20s and 11% said they bought cigarettes less often when buying in packs of 20s.

Just over half of participants (54%) reported that purchasing cigarettes in packs of 25 was better value for money for them compared to just 7% who said purchasing packs of 20s was better value.

See Table 3 for a summary of these results.

### Table 3. Participant responses to the End of Study survey.

|  | n (%) (N = 234) |
| --- | --- |
| **Do you try to make your usual pack of 25 cigarettes last a certain amount of** |  |
| **time?** |  |
| a. Always | 57 (24) |
| b. Sometimes | 124 (53) |
| c. Never | 53 (23) |
| **How long participants try to make their usual pack of 25 last (hours)** (n = 181) mean (SD) | 47.54 (29.16) |
| **Did you try to keep to the same amount of time for pack sizes of 20?** (n = 22)a |  |
| a. Yes | 18 (82) |
| b. No | 4 (18) |
| **Thinking about the two weeks when you were smoking from packs of 20** |  |
| **cigarettes, when you finished a pack, did you usually:** |  |
| a. have another pack ready to start | 196 (84) |
| b. go out to buy another pack | 38 (16) |
| **When did you usually go out to buy another pack?**   1. Immediately, or as soon as I realistically could e.g. first thing in the morning if I finished the pack at night 2. I waited | 35 (92)  3 (8) |
| (n = 38) |  |
| **How long did you typically wait? (hours)**  mean (SD) (n = 3) | 3.67 (2.52) |
| **Thinking about the two weeks when you were smoking from packs of 25** |  |
| **cigarettes, when you finished a pack, did you usually:** |  |
| a. have another pack ready to start | 199 (85) |
| b. go out to buy another pack | 35 (15) |
| **When did you usually go out to buy another pack?**   1. Immediately, or as soon as I realistically could e.g. first thing in the morning if I finished the pack at night 2. I waited | 32 (91)  3 (9) |
| (n = 35) |  |
| **How long did you typically wait? (hours)**  mean (SD) (n = 3) | 4.17 (1.76) |
| **How did you buy your cigarettes when you were buying them in packs of 20?** |  |
| a. in individual packs as I needed them | 51 (22) |
| b. in multiple packs of 20s | 107 (46) |
| c. in a carton containing multiple packs of 20 cigarettes | 68 (29) |
| d. other (please specify) | 8 (3) |
| **How did you buy your cigarettes when you were buying them in packs of 25?** |  |
| a. in individual packs as I needed them | 41 (18) |
| b. in multiple packs of 25 | 114 (49) |

| 1. in a carton containing multiple packs of 25 cigarettes 2. other (please specify) | 67 (29)  12 (5) |
| --- | --- |
| **How often did you buy cigarettes when you were smoking from packs of 20 compared to packs of 25**   1. Less often 2. More often 3. The same | 25 (11)  95 (41)  114 (49) |
| **Which is better value for money for you:** |  |
| a. Buying cigarettes in pack sizes of 20 | 17 (7) |
| b. Buying cigarettes in pack sizes of 25 | 126 (54) |
| c. No difference | 72 (31) |
| d. Don’t know | 19 (8) |
| **Thinking about your experiences of the two cigarette pack sizes (20 and 25),** |  |
| **please indicate in the box below which size you preferred, and why.** |  |
| 20 | 37 (16) |
| 25 | 192 (82) |
| No preference | 5 (2) |
| **Thinking again about your experiences with each cigarette pack size, please** |  |
| **indicate in the box below which size you would prefer to buy from now and** |  |
| **why.** |  |
| 20 | 37 (16) |
| 25 | 190 (81) |
| No preference | 4 (2) |
| Missing | 3 (1) |

**a** Due to an error in Qualtrics survey flow, only participants who answered that they wanted to give the answer to the previous question in hours were shown this question.
